# Prevalence and susceptibility of uropathogens: a recent report from a teaching hospital in Bangladesh

**DOI:** 10.1186/s13104-015-1408-1

**Published:** 2015-09-05

**Authors:** Rezwana Haque, Most. Laila Akter, Md. Abdus Salam

**Affiliations:** Department of Microbiology, Islami Bank Medical College, Rajshahi, Bangladesh; Department of Microbiology, Rajshahi Medical College, Rajshahi, 6000 Bangladesh

**Keywords:** UTI, Uropathogens, Antibiogram, Teaching hospital, Bangladesh

## Abstract

**Background:**

This investigation was aimed to determine the current status of prevalence and antimicrobial susceptibility of uropathogens isolated in a teaching hospital in Bangladesh. A retrospective analysis was done at the department of Microbiology of Islami Bank Medical College, Rajshahi (IBMCR), Bangladesh during January to December, 2012. Midstream clean-catch urine samples were collected from 443 suspected urinary tract infection patients of different age and sex groups. Uropathogens were identified by standard and specific microbiological techniques and antimicrobial susceptibility pattern was determined by Kirby Bauer Disc diffusion method following Clinical and Laboratory Standards Institute (CLSI) guidelines.

**Findings:**

Culture yielded a total of 189 (42.66 %) significant growths of uropathogens including 179 (94.71 %) unimicrobial (single bacterial species) and 10 (5.29 %) polymicrobial (pair of two different bacterial species) growths. Gender distribution showed 34.44 % male and 48.29 % female UTI patients with male to female ratio of 1:1.46, respectively. *E. coli* was the predominant isolate (59.30 %), followed by *Staph saprophyticus* (19.09 %), *Enterococcus* spp. (11.56 %), *Klebsiella* spp. (5.53 %), *Pseudomonas* spp. (2.01 %), *Proteus* spp. (1.51 %) and *Enterobacter* spp. (1.00 %). Very high frequency of resistance ranging from 72.03 to 91.53 % to cotrimoxazole, ciprofloxacin, cefuroxime, cephradin, amoxicillin and nalidixic acid, moderately high resistance to ceftriaxone (55.08 %) and gentamicin (40.68 %) and low resistance to nitrofurantoin (16.10 %) were shown by *E. coli.* Similarly, *Staph. saprophyticus* and *Enterococcus* spp. showed low resistance (18.42 and 21.74 %) to nitrofurantoin, but moderately high against cefaclor, gentamycin, cefuroxime and ceftriaxone. *Klebsiella* spp. and *Proteus* spp. were 72.73 and 66.67 % susceptible, respectively to gentamycin only but low frequency of susceptibility (<50 %) was found to all other antimicrobial agents. *Peudomonas* spp. was 75 % susceptible to nitrofurantoin only and showed 75–100 % resistance to all other agents. *Enterobacter* spp. were 50 % resistant to nitrofurantoin, gentamycin, cefuroxime, cefaclor and ceftriaxone but showed 100 % resistance to all remaining antimicrobials.

**Conclusions:**

Current uropathogens showed the highest rate of susceptibility to nitrofurantoin and gentamicin which can be adapted for empirical treatment of urinary tract infections.

## Background

Urinary tract infection (UTI) remains as one of the most common bacterial infections and second most common infectious disease in community practice with approximately 150 million diagnosed cases each year [1]. Presence of more than 10^5^ organisms per ml in a midstream sample of urine refers to significant bacteriuria and caused mainly by normal bowel flora, *Escherichia coli,* which is responsible for over 75 % of cases [2, 3]. Other members of *Enterobacteriaceae* and a few Gram positive bacteria like *Staphylococcus saprophyticus* and *Enterococcus faecalis* are also responsible for UTI. Usually most of the urinary tract infections are caused by a single bacterial species but polymicrobial infections may also take place [4].

It is estimated that about 35 % of healthy women suffer from symptoms of urinary tract infection at some point in their life. The incidence of UTI is greater in women as compared to men, which may be either due to anatomical predisposition or other host factors [5]. Vaginal colonization with uropathogens precedes most UTIs and sexual activity, pregnancy, obstruction are among the other factors contributing to increasing frequency of UTI in female [6].

Indiscriminate use of antimicrobial agents is a common practice in underdeveloped and many developing countries that often leads to emergence of resistant microorganisms to one or several of these agents with gradual narrowing of scope for effective molecules to combat bacterial infections including UTIs [7]. As a common practice, empirical antimicrobial treatment is initiated before the laboratory results of urine culture are available which may lead to emergence and spread of antimicrobial resistant strains. Factually antimicrobial resistance is one of the principal causes of treatment failure in infectious diseases and a great concern for UTIs [8]. The prevalence and pattern of antimicrobial susceptibility of uropathogens are dependent on many factors and constantly changing with the ever increasing use of antimicrobials, continuous monitoring of the susceptibility pattern is of paramount importance for not only selecting appropriate drugs but also for rational choice of empirical therapy [9]. The present investigation was carried out to determine the recent status of prevalence of bacterial pathogens and their antimicrobial susceptibility in UTI patients with the aim to disseminate information about choice of empirical antibiotics.

## Findings

### Patients

The protocol was approved by the Ethical Review Committee of Islami Bank Medical College, Rajshahi, Bangladesh and informed written consent was taken from patients before collection of their sample. This retrospective analysis included 443 consecutively collected midstream and/or catheter-catch urine samples from clinically suspected patients of UTI of different age and sex attending either at the outpatient department (OPD) or admitted in the Islami Bank Medical College Hospital, Rajshahi, Bangladesh from January to December, 2012. Microscopic demonstration of pus cells >5/HPF (high power field) in a centrifuged deposit of urine was included as study cases [10]. Patients were advised to collect clean-catch midstream or catheter-catch urine into a sterile wide mouth container/test tube with all aseptic measures (information on how to collect proper sample in sterile container aseptically was given prior to collection).

### Urine culture and antibiogram

Urine samples were inoculated aseptically on chromogenic agar (Hicrome UTI agar), Blood agar and MacConkey agar media by using a calibrated wire loop of 28G with an internal diameter of 3.26 mm holding 0.004 ml of urine and incubated overnight at 37 °C aerobically. Details of culture technique and identification methods have been reported previously [11]. Mueller–Hinton agar was used for antimicrobial susceptibility testing (AST) following Kirby–Bauer disc diffusion method [12] against a panel of 11 antibiotics (Oxoid, UK); amoxicillin (10 mcg), nitrofurantoin (300 mcg), cephalexin (30 mcg), cefuroxime (30 mcg), cefaclor (30 mcg), ceftriaxone (30 mcg), ciprofloxacin (10 mcg), gentamicin (10 mcg), nalidixic acid (30 mcg) and co-trimoxazole (25 mcg). As per the Clinical Laboratory Standard Institute (CLSI) guidelines [13], susceptibility was noted as sensitive and resistant based on the diameter of zone of inhibition. *Escherichia coli* ATCC 25922 and *Staphyloccus aureus* ATCC 25923 were used as control strains for interpretations of AST.

### Statistical analysis

All data were entered into Statistical Package for Social Sciences (SPSS) version 16.0. Frequencies with percentage were generated for categorical variables such as, type of bacteria, rate of isolation, and rate of polymicrobial growth in culture.

## Results

A total of 443 cases of different age and sex those who fulfilled the inclusion criteria of suspected UTI were included in this study. Of 443 cases, 180 (40.63 %) were male and 263 (59.37 %) were female with a male to female ratio of 1:1.46. Rate of isolation of uropathogens in male and female was 34.44 and 48.29 %, respectively (Table 1).

**Table 1:** Gender distribution for rate of isolation in urine culture (n = 443)

Sex	No. of sample	Growth of uropathogen n (%)	No growth of uropathogen n (%)
Male	180	62 (34.44)	118 (65.56)
Female	263	127 (48.29)	136 (51.71)
Total	443	189 (42.66)	254 (57.34)

Culture of 443 urine samples yielded a total of 189 (42.66 %) bacterial growths including 179 (94.71 %) unimicrobial (single bacterial species) and 10 (5.29 %) polymicrobial (pair of two different bacterial species) growths. *E. coli* was the predominant isolates 118 (59.30 %), followed by *Staph. saprophyticus* 38 (19.09 %), *Enterococcus* spp. 23 (11.56 %), *Klebsiella* spp. 11 (5.53 %), *Pseudomonas* spp. 04 (2.01 %), *Proteus* spp. 03 (1.51 %) and *Enterobacter* spp. 02 (1.00 %) (Table 2). Polymicrobial growths showed combination of *E. coli* and *Staph. saprophyticus* in 04, *E. coli* and *Enterococci* spp. in 03, *E. coli* and *Klebsiella* spp. in 02 and *E. coli* and *Proteus* spp. in 01 culture-positive plate, respectively.

**Table 2:** Pattern of bacteria isolated from urine culture (n = 199)

Bacteria	Number	Percentage
*E. coli*	118	59.30
*Staph. saprophyticus*	38	19.09
*Enterococcus* spp.	23	11.56
*Klebsiella* spp.	11	05.53
*Pseudomonas* spp.	04	02.01
*Proteus* spp.	03	01.51
*Enterobacter* spp.	02	01.00
Total	199	100

N.B. (Nota Bene): 189 culture positive samples of urine yielded 199 bacterial isolates including both single (179) and polymicrobial growths (10) of two bacteria each

The antimicrobial resistance patterns of isolates are shown in Table 3. Very high frequency of resistance ranging from 72.03 to 91.53 % to cotrimoxazole, ciprofloxacin, cefuroxime, cephradin, amoxicillin and nalidixic acid, moderately high resistance to ceftriaxone (55.08 %) and gentamicin (40.68 %) and low resistance to nitrofurantoin (16.10 %) were shown by *E. coli.* Similarly, *Staph. saprophyticus* and *Enterococcus* spp. showed low resistance (18.42 and 21.74 %) to nitrofurantoin, but moderately high against cefaclor, gentamycin, cefuroxime and ceftriaxone. *Klebsiella* spp. and *Proteus* spp. were 72.73 and 66.67 % susceptible, respectively to gentamycin only but low frequency of susceptibility (<50 %) was found to all other antimicrobial agents. *Peudomonas* spp. was 75 % susceptible to nitrofurantoin only and showed 75–100 % resistance to all other agents. *Enterobacter* spp. were 50 % resistant to nitrofurantoin, gentamycin, cefuroxime, cefaclor and ceftriaxone but showed 100 % resistance to all remaining antimicrobials.

**Table 3:** Antimicrobial resistance pattern of uropathogens (n = 199)

Antimicrobial agent	*E. coli* (n = 118)	*Staph. saprophyticus* (n = 38)	*Enterococcus* spp. (n = 23)	*Klebsiella* spp. (n = 11)	*Pseudomonas* spp. (n = 04)	*Proteus* spp. (n = 03)	*Enterobacter* spp. (n = 02)
	R %	R %	R %	R %	R %	R %	R %
Amoxicillin	89.83	71.05	60.87	90.91	100	100	100
Nitrofurantoin	16.10	18.42	21.74	63.64	25	66.67	50
Cephalexin	80.51	65.79	78.26	100	100	100	100
Cefuroxime	78.81	39.47	60.87	63.64	100	100	50
Cefaclor	60.17	73.68	78.26	72.73	100	100	50
Ceftriaxone	55.08	44.74	47.83	54.55	75	66.67	50
Ciprofloxacin	72.88	63.16	82.61	81.82	100	100	100
Gentamicin	40.68	47.37	56.52	27.27	75	33.33	50
Nalidixic acid	91.53	92.11	95.65	100	100	100	100
Co-trimoxazole	72.03	73.68	73.91	72.73	100	100	100

N.B. (Nota Bene): *R* resistance

## Discussion

Urinary tract infection is emerging as an important community acquired and nosocomial bacterial infection. Moreover, antimicrobial resistance to various classes of antimicrobials to uropathogens continues to be a major health problem in different parts of the world [14, 15]. In the present setting, rate of isolation of 59.67 % including 94.41 % unimicrobial and 5.59 % polymicrobial growths corroborates well with a few reports from Bangladesh, India and in Pakistan [16–18].

It is documented that UTI is more common in females than in males and findings of our investigation are also in agreement with this generalization and rightly coincided with a recent study done by Deshpande et el. [19]. Likewise, regarding prevalence of uropathogens, our observation is in good agreement with several previous reports [16, 19, 20].

Increasing drug resistance is a great concern to common bacterial infections including UTI. Still antimicrobial agents like amoxicillin, cotrimoxazole, cephradin, nalidixic acid, ciprofloxacin, azithromycin are in place to treat many gram-positive and gram-negative bacterial infections including UTI in many underdeveloped and developing countries including Bangladesh. Unfortunately all these agents were found to have unacceptable range of antimicrobial activity to uropathogens isolated in our setting. This finding is alarming in regards to the choice of effective therapeutic options in the treatment of UTI and obviously a great concern to treating physicians. Ciprofloxacin was once considered to be the drug of choice for uncomplicated and complicated UTI but due to lack of rational use, this broad spectrum molecule has entirely lost its efficacy not only in UTI but to other common infections too. Similar picture is also noted in case of 1st, 2nd and 3rd generations cephalosporin. It is reasonable to speculate that there were a few cases of ESBL-producing uropathogens especially from Gram-negative isolates that couldn’t be separated in the present investigation due to limitation are thought to be responsible for resistance to different generations of cephalosporin. Nitrofurantoin was found to be reasonably high efficacious agent among all antimicrobials used to almost all uropathogens in the current setting and similar results were also reported from other studies [21–23]. This is good news indeed especially for uncomplicated UTI and prophylaxis in the context of gradually decreasing susceptibility of most of the comparatively cheaper oral anti-UTI drugs. Though moderate to high susceptibility was also noted for gentamicin and ceftriaxone for most of the uropathogens which is comparable to nitrofurantoin but one has to remember that their uses are limited due to parenteral route and patient’s noncompliance.

Among gram-negative isolates, *Pseudomonas* spp. is most famous for hospital acquired UTI and conventional antimicrobials are usually ineffective against *Pseudomonas* infections. Though nitrofurantoin was found to have high frequency of susceptibility against *Pseudomonas* UTI but it is only indicated in uncomplicated UTI or for prophylaxis. In the recent years, though precious drugs like carbapenems are being used for Pseudomonad infections but in order to preserve its long term efficacy, we recommend that its use should be restricted to special circumstances.

We appreciate some shortcomings of our work in the context of lacking of clinical information. This study was based on retrospective laboratory data only so we failed to provide information on categorization of UTI patients whether symptomatic or asymptomatic, complicated or uncomplicated. Further, distribution of patients based on the sources of infection like catheter-associated, community acquired or nosocomial also could not be mentioned.

## Conclusions

Though pattern of uropathogens doesn’t vary too much in different settings but increasing antimicrobial resistance to bacteria causing UTI is a great concern all over and under developed and developing countries in particular. Still, nitrofurantoin holds much optimism in treating uncomplicated UTI but there is no alternative to rational use of antibiotics to preserve long term efficacy of many excellent molecules including nitrofurantoin. Selection of antimicrobials for UTI should be guided by culture and sensitivity and empirical therapy must be considered on the recent antibiogram of a particular geographical area.

## References

[1] Akram M, Shahid M, Khan AU (2007). Etiology and antibiotic resistance patterns of community-acquired urinary tract infections in J N M C Hospital Aligarh, India. Ann Clin Microbiol Antimicrob.

[2] Goddard J, Turner AN, Cumming AD, Stewart LH, Boon NA, Colledge NR, Walker BR, Hunter JAA (2006). Kidney and urinary tract disease. Davidson’s principles and practice of Medicine.

[3] Lee LB, Neild HG (2007). Urinary tract infection. Medicine.

[4] Ahmed S, Rashid HU (1996). Urinary tract infection in adults—a review. Bangladesh Ren J.

[5] Schaeffer AJ, Rajan N, Cao Q, Anderson BE (2001). Host pathogenesis in urinary tract infection. Int J Antimicrob Agents..

[6] Stamm WE, Kasper DL, Braunwald E, Fausi AS (2005). Urinary tract infections and pyeloephritis. Harrison’s principles of internal medicine.

[7] Gold HS, Moellering RC (1996). Antimicrobial drug resistance. N Eng J Med.

[8] Gupta K (2002). Addressing antibiotic resistance. Am J Med.

[9] Bauza E, Cercenado E (2002). *Klebsiella* and *Enterobacter* antibiotic resistance and treatment implications. Semin Respis Infect.

[10] Fuller CE, Threatte GA, Henry JB, Henry JB, Davey FR, Herman CJ, McPherson RA, Pincus MR, Threatte GA, Woods GL (2001). Basic examination of urine. Clinical diagnosis and management by laboratory methods.

[11] Akter ML, Haque R, Salam MA (2014). Comparative evaluation of chromogenic agar medium and conventional culture system for isolation and presumptive identification of uropathogens. Pak J Med Sci..

[12] Bauer AW, Kirby WMM, Sherris JC, Turek M (1966). Antibiotic susceptibility testing by a standardized single disc method. Am J Clin Pathol.

[13] Clinical and Laboratory Standard Institute. Performance standards for antimicrobial susceptibility testing: sixteenth informational supplement 2006. CLSI document M100-S16 CLSI, Wayne, PA.

[14] Oliveira FA, Paludo KS, Arend LNVS, Farah SMSS, Pedrosa FO, Souza EM, Surek M, Picheth G, Fadel-Picheth CMT (2011). Virulence characteristics and antimicrobial susceptibility of uropathogenic *Escherichia coli* strains. Genet Mol Res.

[15] Farshad S, Ranjbar R, Japoni A, Hosseini M, Anvarinejad M, Mohammadzadegan R (2012). Microbial susceptibility, virulence factors and plasmid profiles of uropathogenic *Escherichia coli* strains isolated from children in Jahron. Iran. Arch Iran Med.

[16] Qaiser S, Zeeshan M, Jabeen K, Ahsan T, Zafar A (2011). Comparison of chromogenic urinary tract infection medium with cysteine lactose electrolyte deficient media in a resource limited setting. J Pak Med Assoc.

[17] Parveen R, Saha SK, Shamshuzzaman SM, Rashid AL, Chowdhury A, Muazzam N (2011). Detection of uropathogens by using chromogenic media (Hicrome UTI agar), CLED agar and other conventional media. Faridpur Med Coll J.

[18] Rani L, Pinnelli VBK, Hemavathi, Belwadi S, Rajendran R (2012). Utility Urinary of HiChrome Tract Infection (UTI) agar medium for identification of uropathogens: a comparative study with other conventional media. J Chem Pharm Res.

[19] Deshpande KD, Pichare AP, Suryawanshi NM, Davane MS (2011). Antibiogram of gram negative uropathogens in hospitalized patients. Int J Recent Trends Sci Technol.

[20] Keah S, Wee E, Chng K (2007). Antimicrobial susceptibility of community-acquired uropathogens in general practice. Malays Fam Phys.

[21] Sharifian M, Karimi A, Rafiee-Tabatabaei S (2006). Microbial sensitivity pattern in urinary tract infections in children: a single center experience of 1177 urine cultures. Jpn J Infect Dis.

[22] Kothari A, Sagar V (2008). Antibiotic resistance in pathogens causing community-acquired urinary tract infections in India: a multicenter study. J Infect Dev Ctries.

[23] Sohely S, Farhana AF, Fahmida, Saleh AA (2009). Sensitivity pattern of uropathogens in children. Bangladesh J Med Microbiol.

